# *trans*-Dibromidobis(1-ethyl-3-methyl­imidazol-2-yl­idene)palladium(II)

**DOI:** 10.1107/S1600536811030480

**Published:** 2011-08-06

**Authors:** Solveig R. Madsen, Nina Lock, Jacob Overgaard, Bo B. Iversen

**Affiliations:** a Department of Chemistry & Center for Materials Crystallography, Aarhus University, Aarhus, Denmark

## Abstract

The title compound, *trans*-[PdBr_2_(C_6_H_10_N_2_)_2_], was synthesized ionothermally in the ionic liquid solvent 1-ethyl-3-methyl­imidazolium bromide. In the crystal, the Pd^II^ atoms are square-planarly coordinated to two Br atoms and two neutral (C_6_H_10_N_2_) ligands. The Pd^II^ atom is located on an inversion centre.

## Related literature

The title complex shares many features with a number of known structures, which also contain a Pd^II^ atom square-planarly coordinated to two bromide ligands in *trans*-conformation as well as two equivalent organic ligands (Hahn *et al.*, 2004 ▶; Huynh & Wu, 2009 ▶). A few of these structures even have the same space group and in some structures the organic ligand is also an imidazolium derivative (Dash *et al.*, 2010 ▶). The title compound was obtained in a attempt to simplify the synthesis of the *cis*-complex which was described previously (Madsen *et al.*, 2011 ▶). For information on the ionothermal synthesis method, see: Welton (1999 ▶); Babai & Mudring (2006 ▶); Morris (2009 ▶).
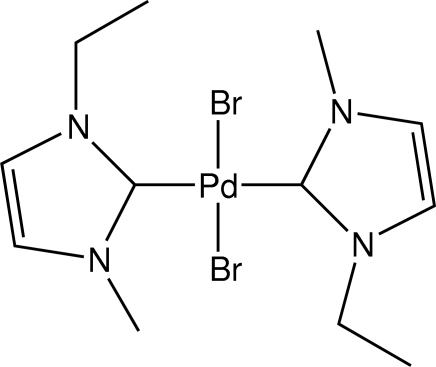


## Experimental

### 

#### Crystal data

[PdBr_2_(C_6_H_10_N_2_)_2_]
*M* *_r_* = 486.54
Monoclinic, 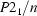

*a* = 8.3093 (2) Å
*b* = 8.6868 (2) Å
*c* = 12.0788 (3) Å
β = 101.741 (1)°
*V* = 853.62 (4) Å^3^
*Z* = 2
Mo *K*α radiation
μ = 5.76 mm^−1^
*T* = 296 K
0.15 × 0.15 × 0.1 mm

#### Data collection

Bruker X8 APEXII diffractometer
Absorption correction: multi-scan (*SADABS*; Sheldrick, 2008*a* ▶) *T*_min_ = 0.585, *T*_max_ = 0.711
27485 measured reflections
2582 independent reflections
1894 reflections with *I* > 2σ(*I*)
*R*_int_ = 0.022

#### Refinement

*R*[*F* ^2^ > 2σ(*F* ^2^)] = 0.044
*wR*(*F* ^2^) = 0.167
*S* = 1.63
2582 reflections
88 parameters
H-atom parameters constrained
Δρ_max_ = 1.84 e Å^−3^
Δρ_min_ = −1.02 e Å^−3^

### 

Data collection: *APEX2* (Bruker, 2011 ▶); cell refinement: *SAINT* (Bruker, 2011 ▶); data reduction: *SAINT*; program(s) used to solve structure: *SHELXS97* (Sheldrick, 2008*b* ▶); program(s) used to refine structure: *SHELXL97* (Sheldrick, 2008*b* ▶); molecular graphics: *ORTEP-3 for Windows* (Farrugia, 1997 ▶) and *Mercury* (Macrae *et al.*, 2006 ▶); software used to prepare material for publication: *WinGX* (Farrugia, 1999 ▶).

## Supplementary Material

Crystal structure: contains datablock(s) global, I. DOI: 10.1107/S1600536811030480/zk2019sup1.cif

Structure factors: contains datablock(s) I. DOI: 10.1107/S1600536811030480/zk2019Isup2.hkl

Additional supplementary materials:  crystallographic information; 3D view; checkCIF report

## Figures and Tables

**Table:** 

C1—Pd1	2.023 (4)
Br1—Pd1	2.4364 (5)

**Table:** 

C1—Pd1—C1^i^	180
C1—Pd1—Br1	89.14 (12)
C1^i^—Pd1—Br1	90.86 (12)
Br1—Pd1—Br1^i^	180

Symmetry code: (i) 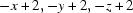.

**Fig. 1.**
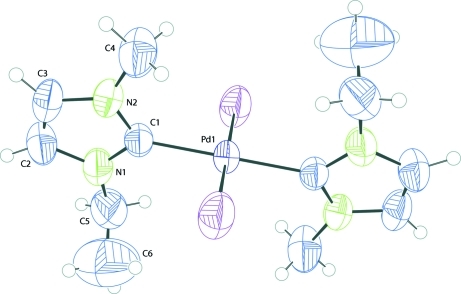
The molecular structure of trans-[PdBr2(C6H10N2)2], with atom labels and 50% probability displacement ellipsoids for non-H atoms.

## supplementary crystallographic information

### Experimental

The title compound was obtained when palladium(II) acetate and 1-ethyl-3- methylimidazolium bromide were mixed and heated in an autoclave at 100°C for 8 days.

### Refinement

All H atoms were positioned geometrically and refined using a riding model with C—H = 0.95–0.99 Å and with *U*_iso_(H) = 1.2 times *U*_eq_(C).

### Crystal data

**Table:**

[PdBr_2_(C_6_H_10_N_2_)_2_]	*F*(000) = 472
*M**_r_* = 486.54	*D*_x_ = 1.893 Mg m^−^^3^
Monoclinic, *P*2_1_/*n*	Mo *K*α radiation, λ = 0.71073 Å
Hall symbol: -P 2yn	Cell parameters from 9872 reflections
*a* = 8.3093 (2) Å	θ = 5.5–54.7°
*b* = 8.6868 (2) Å	µ = 5.76 mm^−^^1^
*c* = 12.0788 (3) Å	*T* = 296 K
β = 101.741 (1)°	Square, colourless
*V* = 853.62 (4) Å^3^	0.15 × 0.15 × 0.1 mm
*Z* = 2	

### Data collection

**Table:**

Bruker X8 APEXII diffractometer	2582 independent reflections
Radiation source: fine-focus sealed tube	1894 reflections with *I* > 2σ(*I*)
graphite	*R*_int_ = 0.022
Detector resolution: 12.00 pixels mm^-1^	θ_max_ = 30.5°, θ_min_ = 2.7°
Narrow slices collected using φ and ω scans	*h* = −11→11
Absorption correction: multi-scan (*SADABS*; Sheldrick, 2008a)	*k* = −12→12
*T*_min_ = 0.585, *T*_max_ = 0.711	*l* = −15→17
27485 measured reflections	

### Refinement

**Table:**

Refinement on *F*^2^	Primary atom site location: structure-invariant direct methods
Least-squares matrix: full	Secondary atom site location: difference Fourier map
*R*[*F*^2^ > 2σ(*F*^2^)] = 0.044	Hydrogen site location: inferred from neighbouring sites
*wR*(*F*^2^) = 0.167	H-atom parameters constrained
*S* = 1.63	*w* = 1/[σ^2^(*F*_o_^2^) + (0.0705*P*)^2^] where *P* = (*F*_o_^2^ + 2*F*_c_^2^)/3
2582 reflections	(Δ/σ)_max_ = 0.008
88 parameters	Δρ_max_ = 1.84 e Å^−^^3^
0 restraints	Δρ_min_ = −1.02 e Å^−^^3^

### Special details

**Table:**

Geometry. All e.s.d.'s (except the e.s.d. in the dihedral angle between two l.s. planes) are estimated using the full covariance matrix. The cell e.s.d.'s are taken into account individually in the estimation of e.s.d.'s in distances, angles and torsion angles; correlations between e.s.d.'s in cell parameters are only used when they are defined by crystal symmetry. An approximate (isotropic) treatment of cell e.s.d.'s is used for estimating e.s.d.'s involving l.s. planes.
Refinement. Refinement of *F*^2^ against ALL reflections. The weighted *R*-factor *wR* and goodness of fit *S* are based on *F*^2^, conventional *R*-factors *R* are based on *F*, with *F* set to zero for negative *F*^2^. The threshold expression of *F*^2^ > σ(*F*^2^) is used only for calculating *R*-factors(gt) *etc*. and is not relevant to the choice of reflections for refinement. *R*-factors based on *F*^2^ are statistically about twice as large as those based on *F*, and *R*- factors based on ALL data will be even larger.

### Fractional atomic coordinates and isotropic or equivalent isotropic displacement parameters (Å^2^)

**Table:**

	*x*	*y*	*z*	*U*_iso_*/*U*_eq_	
N2	0.8710 (5)	0.9917 (3)	0.7471 (3)	0.0598 (9)	
C1	0.9896 (5)	0.9475 (5)	0.8355 (3)	0.0570 (8)	
C3	0.9059 (7)	0.9436 (6)	0.6478 (4)	0.0735 (11)	
H3	0.8411	0.9594	0.5763	0.088*	
C5	1.2461 (7)	0.7839 (10)	0.8505 (6)	0.118 (2)	
H5A	1.2269	0.747	0.9225	0.142*	
H5B	1.2675	0.6958	0.8062	0.142*	
C2	1.0451 (6)	0.8718 (7)	0.6700 (4)	0.0852 (14)	
H2	1.1002	0.8294	0.6176	0.102*	
C6	1.3777 (13)	0.8815 (14)	0.8675 (10)	0.197 (5)	
H6A	1.473	0.8289	0.9084	0.295*	
H6B	1.3548	0.9691	0.9104	0.295*	
H6C	1.3977	0.9152	0.7959	0.295*	
C4	0.7242 (7)	1.0768 (7)	0.7604 (5)	0.0953 (17)	
H4A	0.6572	1.0973	0.6873	0.143*	
H4B	0.7563	1.1723	0.7986	0.143*	
H4C	0.6629	1.0166	0.8041	0.143*	
Br1	1.09068 (7)	1.25854 (6)	0.96387 (4)	0.0893 (2)	
N1	1.0978 (4)	0.8699 (5)	0.7886 (3)	0.0772 (10)	
Pd1	1	1	1	0.0555 (2)	

### Atomic displacement parameters (Å^2^)

**Table:**

	*U*^11^	*U*^22^	*U*^33^	*U*^12^	*U*^13^	*U*^23^
N2	0.075 (2)	0.060 (2)	0.0438 (19)	0.0033 (13)	0.0110 (16)	0.0020 (13)
C1	0.064 (2)	0.063 (2)	0.047 (2)	−0.0089 (17)	0.0168 (16)	−0.0047 (18)
C3	0.104 (4)	0.073 (3)	0.044 (2)	−0.009 (3)	0.016 (2)	−0.002 (2)
C5	0.080 (3)	0.189 (7)	0.088 (4)	0.017 (4)	0.023 (3)	−0.011 (4)
C2	0.100 (4)	0.104 (4)	0.059 (3)	−0.001 (3)	0.033 (3)	−0.006 (3)
C6	0.136 (7)	0.207 (12)	0.231 (13)	−0.009 (9)	0.000 (7)	0.036 (9)
C4	0.104 (4)	0.114 (4)	0.064 (3)	0.052 (4)	0.010 (3)	0.006 (3)
Br1	0.1261 (5)	0.0831 (4)	0.0624 (4)	−0.0312 (3)	0.0278 (3)	−0.0070 (2)
N1	0.0732 (19)	0.108 (3)	0.0529 (19)	0.008 (2)	0.0181 (16)	−0.0060 (19)
Pd1	0.0597 (3)	0.0696 (4)	0.0386 (3)	−0.00208 (16)	0.01357 (18)	−0.00364 (15)

### Geometric parameters (Å, °)

**Table:**

N2—C1	1.353 (6)	C2—N1	1.410 (6)
N2—C3	1.356 (6)	C2—H2	0.93
N2—C4	1.463 (6)	C6—H6A	0.96
C1—N1	1.338 (5)	C6—H6B	0.96
C1—Pd1	2.023 (4)	C6—H6C	0.96
C3—C2	1.293 (7)	C4—H4A	0.96
C3—H3	0.93	C4—H4B	0.96
C5—C6	1.366 (12)	C4—H4C	0.96
C5—N1	1.503 (7)	Br1—Pd1	2.4364 (5)
C5—H5A	0.97	Pd1—C1^i^	2.023 (4)
C5—H5B	0.97	Pd1—Br1^i^	2.4364 (5)
			
C1—N2—C3	111.0 (4)	H6A—C6—H6B	109.5
C1—N2—C4	123.1 (4)	C5—C6—H6C	109.5
C3—N2—C4	125.9 (4)	H6A—C6—H6C	109.5
N1—C1—N2	104.7 (3)	H6B—C6—H6C	109.5
N1—C1—Pd1	129.1 (3)	N2—C4—H4A	109.5
N2—C1—Pd1	126.2 (3)	N2—C4—H4B	109.5
C2—C3—N2	107.9 (4)	H4A—C4—H4B	109.5
C2—C3—H3	126	N2—C4—H4C	109.5
N2—C3—H3	126	H4A—C4—H4C	109.5
C6—C5—N1	108.5 (8)	H4B—C4—H4C	109.5
C6—C5—H5A	110	C1—N1—C2	109.1 (4)
N1—C5—H5A	110	C1—N1—C5	126.4 (4)
C6—C5—H5B	110	C2—N1—C5	124.3 (4)
N1—C5—H5B	110	C1—Pd1—C1^i^	180.000 (2)
H5A—C5—H5B	108.4	C1—Pd1—Br1	89.14 (12)
C3—C2—N1	107.2 (4)	C1^i^—Pd1—Br1	90.86 (12)
C3—C2—H2	126.4	C1—Pd1—Br1^i^	90.86 (12)
N1—C2—H2	126.4	C1^i^—Pd1—Br1^i^	89.14 (12)
C5—C6—H6A	109.5	Br1—Pd1—Br1^i^	180
C5—C6—H6B	109.5		
			
C3—N2—C1—N1	−0.5 (5)	Pd1—C1—N1—C5	8.6 (7)
C4—N2—C1—N1	178.1 (4)	C3—C2—N1—C1	−2.4 (6)
C3—N2—C1—Pd1	176.8 (3)	C3—C2—N1—C5	173.7 (5)
C4—N2—C1—Pd1	−4.7 (6)	C6—C5—N1—C1	−91.6 (8)
C1—N2—C3—C2	−1.1 (6)	C6—C5—N1—C2	93.0 (8)
C4—N2—C3—C2	−179.6 (5)	N1—C1—Pd1—Br1	100.3 (4)
N2—C3—C2—N1	2.1 (6)	N2—C1—Pd1—Br1	−76.2 (4)
N2—C1—N1—C2	1.7 (5)	N1—C1—Pd1—Br1^i^	−79.7 (4)
Pd1—C1—N1—C2	−175.4 (3)	N2—C1—Pd1—Br1^i^	103.8 (4)
N2—C1—N1—C5	−174.3 (5)		

Symmetry codes: (i) −*x*+2, −*y*+2, −*z*+2.
